# Rehabilitation after first-time anterior cruciate ligament injury and reconstruction in female football players: a study of resilience factors

**DOI:** 10.1186/s13102-016-0046-9

**Published:** 2016-07-16

**Authors:** Urban Johnson, Andreas Ivarsson, Jón Karlsson, Martin Hägglund, Markus Waldén, Mats Börjesson

**Affiliations:** Center of Research on Welfare, Health and Sport, Halmstad University, Box 823, S-301 18 Halmstad, Sweden; Department of Orthopaedics, Sahlgrenska University Hospital, Gothenburg, S-413 45 Sweden; Sahlgrenska Academy Institute of Clinical Sciences, Gothenburg University, Gothenburg, S-405 30 Sweden; Football Research Group, Linköping, Sweden; Department of Medical and Health Sciences, Division of Physiotherapy, Linköping University, Linköping, S-581 83 Sweden; Department of Medical and Health Sciences, Division of Community Medicine, Linköping University, Linköping, S-581 83 Sweden; The Swedish School of Sport and Health Sciences, Stockholm, Box 5626, S-114 86 Sweden; Karolinska University Hospital, Stockholm, S-171 77 Sweden

**Keywords:** Athletic injury, Behavior, Female football, Psychosocial, Resilience

## Abstract

**Background:**

Most of the research in the area of psychosocial factors in rehabilitation after sports injuries has focused on risk behaviors, while relatively few studies have focused on behaviors that facilitate rehabilitation. The objective of our study was to understand the psychosocial features that characterize elite female football players who express a resilient behaviour during rehabilitation after a first-time anterior cruciate ligament (ACL) injury and reconstruction.

**Methods:**

A qualitative method was used based on individual in-person interviews and video communication of players who incurred a first-time ACL tear during the 2012 season of the Swedish Women’s Elite Football League. In total, 13 players had a first-time ACL and were interviewed post-season. The interviews were followed by a thematic content analysis. Based on this, eight players were identified as showing resilient behaviors during their rehabilitation and were included in the final analysis.

**Results:**

Three core themes representing psychosocial factors that help players cope successfully with rehabilitation were identified: (I) constructive communication and rich interaction with significant others; (II) strong belief in the importance and efficacy of one’s own actions; and (III) the ability to set reasonable goals.

**Conclusions:**

The findings suggest three core themes of psychosocial factors that characterize first-time ACL-injured elite female football players showing resilience during rehabilitation after ACL reconstruction. Suggestions for medical teams about ways to support communication, self-efficacy, and goal-setting during the rehabilitation process, are provided.

## Background

Anterior cruciate ligament (ACL) injury is a major problem in sport, especially in women’s team sports such as football [1]. The rehabilitation period is long and demanding and the average time to return to play in women’s football is about 8.5 months or longer [2]. Negative mood disturbance, reduced self-confidence, and fear of re-injury may be experienced by ACL-injured athletes resulting in a lower return to sporting activity [3, 4]. The majority of previously published research in the area of psychosocial factors during rehabilitation after ACL injury has focused on risk factors that might inhibit a successful return to play, such as fear of re-injury [5] and external locus of control [6]. In contrast, only a few studies have primarily focused on psychosocial factors facilitating athletes’ rehabilitation [5]. Nevertheless, factors previously associated with more successful rehabilitation after injury, in sports such as football and rugby, includes setting goals and objectives during rehabilitation [7], belief in the efficacy of treatment [8], rehabilitation practitioner expectations of patient adherence [9], social support [10], and constructive communication [11].

*Resilience* is a dynamic capability that helps people strive to realize their goals [12, 13]. The dimensions of resilience, which include self-efficacy, self-control, hardiness, ability to engage support and help, learning from difficulties, social problem-solving, and persistence despite obstacles to progress, are all recognized as qualities that are important for positive experiences and that, together with adaptive behaviors during rehabilitation, will increase the chance for positive outcomes in relation to severe injuries [14, 15]. In a study of 32 recovered knee- and ankle-injured athletes (mean overall recovery time of 10 weeks), the largest differences between the fastest and slowest “healers” were found related to three variables [16]. Fast healers used more goal setting, positive self-talk, and healing imagery than slow healers. These results support the idea that certain attitudes and psychosocial factors may enhance the effectiveness of particular treatments, as well as an injured athlete’s ability to cope. The results from a more recent correlational study showed that problem-focused coping strategies aimed at improving autonomy and confidence were psychologically beneficial for ACL-injured professional rugby union players in terms of enhanced well-being [17]. In a systematic review of the psychological factors associated with returning to sport following injury [18], positive psychological responses, including high levels of motivation and confidence, were associated with a greater likelihood of returning to the athletes’ pre-injury levels of participation. In addition, a high internal health locus of control and high self-efficacy were useful cognitive factors to master ACL injury rehabilitation, together with a low level of fear of re-injury. Moreover, athletes who successfully returned to sports were more experienced and more established players compared to those who did not return to sports after their injuries [18]. Taken together, the existing literature points out several psychosocial factors that may help players cope successfully with rehabilitation. However, to the best of our knowledge, no study has examined these issues in a homogeneous sample of first-time ACL-injured elite female football players.

Based on a social constructivist narrative theory, the objectives of this study were to understand the psychosocial variables that characterize players who express a resilient behaviour during rehabilitation after a first-time ACL injury and subsequent reconstruction.

## Methods

### Setting and research design

The participants of this study were identified through a prospective injury surveillance audit carried out in the Swedish Women’s Elite Football League in the 2012 season. The prospective study followed the same design as that previously reported in a similar setting [19]. The Women’s Elite Football League consists of 12 teams with approximately 250 players. During the season 2012, 13 of these players sustained a first-time total ACL tear (all underwent reconstructive surgery) and were approached for inclusion in this study.

We then adopted a two-step design, the first step using an expert evaluation process [20, 21] to identify resilient players (among the 13) based on players’ descriptions of being injured. This was conducted based on interview transcripts and no specific criteria were developed for separating resilient injured players from other players before the start of analyses. The primary goal of this procedure was to obtain a sub-sample of cases from which further data could be extracted [21]. This selection was done through the expert evaluation and the psychosocial profiles being obtained. The players who reported a mixture of several adaptive and maladaptive behaviors and emotions during rehabilitation, such as engaging in primarily emotion-focused strategies, an inability to accept the situation of being severely injured, and expressing worries and concern about the rehabilitation process, were excluded from further analyses.

In the second step, we performed a thematic content analysis (for more information, see Content analysis) focusing only on those players who were identified as showing resilient behaviors during rehabilitation (*n* = 8). In line with the above description of the dimensions of resilience, injured players who demonstrated high self-efficacy as well as high social problem-solving abilities and adaptive behaviors, such as actively working to set reasonable goals during rehabilitation, were identified as resilient players. Their interview data was included into the thematic content analysis.

### Participants

The ages of the eight players included in the thematic content analysis ranged from 25 to 35 years (*M* = 28.0, *SD* = 3.9). The average length of time between the day of the injury and the day of the interview was 6.3 months (SD 2.9) for the resilience group. Players returned to practice approximately 9, 5 months after injury occurred, which parallels data of comparable study groups [2]. In Table 1 description of the players are presented including information on the clinical outcome.

**Table 1:** Description of ACL first time injured female soccer players. Description of the participants

Case	Playing position	Injury type^a^	Surgery	Rehabilitation outcome (month)
#1	Midfielder	Index	Yes	12
#2	Forward	Index	Yes	7
#3	Defender	Index	Yes	10
#4	Midfielder	Index	Yes	10
#5	Midfielder	Index	Yes	12
#6	Forward	Index	Yes	NA^b^
#7	Forward	Index	Yes	6
#8	Defender	Index	Yes	7

^a^Index denotes a first-time injury

^b^Data not available

### Interview guide

The interview guide, consisting of four broad questions (see below), was designed to gather information about the players’ rehabilitation period. We used an open-ended, low-structured interview guide with questions covering a broad area of what happened in their sport and in their lives during the rehabilitation period, including their physiological and emotional states.

The players were asked to: (1) provide background information (i.e., age, years in professional football); (2) describe the event when the injury occurred; (3) tell their stories of how they experienced their situations during rehabilitation from the ACL injury and reconstructive surgery; and (4) describe, as fully as possible, the ways in which they felt their life situation influenced their behaviors during rehabilitation. Additional questions and probes were asked in order to provide richness to the stories.

### Procedure

The time and place of the interviews were scheduled for the post-season (December 2012 to January 2013), and took place 6 months (range 4–9) post reconstruction. All players were located in Sweden at the time of the interviews. The first interview was intended to be a pilot interview to test the interview guide. Because no subsequent changes were made to the interview guide, this interview was included in the study. The second author is well acquainted in qualitative methodology and especially in social constructivist narrative theory [22], which allowed us to recapture the way in which selves and identities were grounded in cultural forms of language and sense-making. The second author conducted all the interviews, lasting 25–65 min and transcribed verbatim all the interviews prior to analysis. The study was reviewed by the Regional Ethical Review Board, Linköping University, Sweden (# M240-09). Informed consent was obtained from all participating players.

### Content analysis

In the second step of the analysis, a thematic content analysis of the resilience group data (*n* = 8) was conducted [23, 24]. The analysis was performed in several steps.

1. The verbatim transcription of each interview was read several times to provide a sense of the whole story, as told by each athlete.
2. The text was then divided into units of meaning, consisting of either several words in a sentence or several sentences bound together by their contents. These units are represented by excerpts such as: “received loads of social support from different persons,” “new perspectives and great hope for the rehabilitation to come,” and “I have several goals and objectives for the future”.
3. The units of meaning were condensed while retaining their original essence and were labelled with codes that simply stated the contents of each. For example, “I have learned from mistakes,” “the head coach encouraged me a lot,” and “use my whole contact network”.
4. To find similarities and differences between the different codes, the codes were compared and sorted into different sub-themes such as “supportive partners outside soccer,” “acceptance of injury,” and “confident thinking”.
5. The sub-themes were then compared and sorted into larger themes that represented different clusters of story elements—for example, “communication outside sports” and “self-determined goal motivation”.
6. Finally, the themes were combined into higher order core themes illustrating the central interpreted meanings of the athletes’ narratives of psychosocial resilience factors facilitating injury rehabilitation (see Table 2).

**Table 2:**
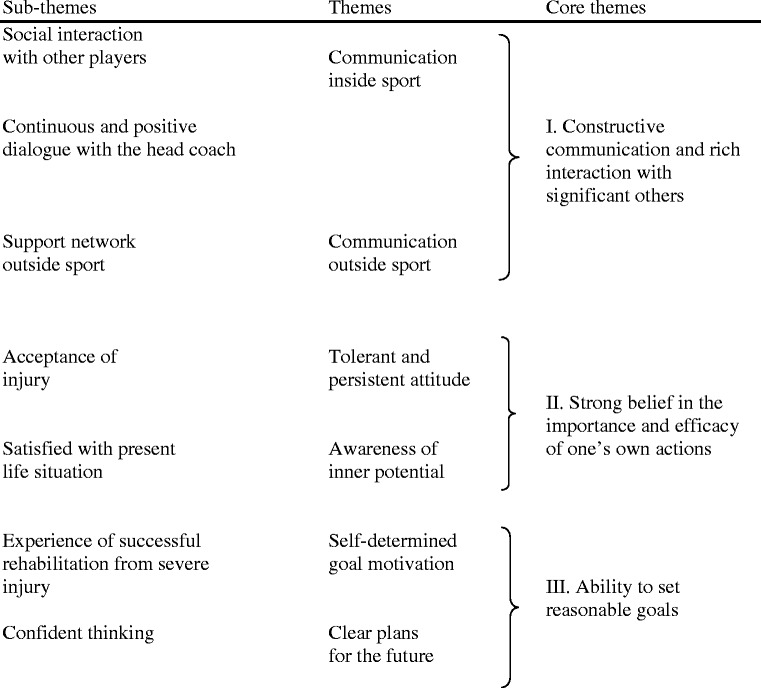
Psychological characteristics of resilient players during rehabilitation after their first ACL injury

To deal with credibility, one aspect of trustworthiness in qualitative studies [25], the authors repeatedly read through the interview transcripts while comparing and validating them against the sub-themes, themes, and core themes, ensuring that no relevant data had been inadvertently or systematically excluded and that no irrelevant data had been included. The authors read and re-read the transcripts throughout the data analysis process. Moreover, another expert in sport and exercise psychology and highly trained in qualitative research was invited to triangulate the steps outlined in the data analysis (1–6). The authors first introduced the verbatim transcriptions of each interview, after which the additional colleague was asked to comment on the condensed statements, codes, sub-themes, themes, and core themes. After several discussions comparing and sorting sub-themes, themes, and core themes in logical order, suggestions were recommended and changes were made accordingly once consensus had been reached.

## Results

The eight *resilient* players experienced adaptive behaviors and emotions and felt that their successful rehabilitation (defined as return to train and play), in many respects, constituted positive learning experiences. Most of the resilient players had well-established positions in their teams and had experienced several previous injuries (but no ACL injury) in their careers.

### Thematic content analysis of identified resilience group

The players’ narratives indicated numerous behaviors inside and outside sport: tolerant and persistent attitude, awareness of inner potential, self-determined goal motivation, and clear plans for the future. The different psychosocial aspects of the narratives, each with its own unique structure, are presented separately, along with themes and underlying sub-themes. Below, we primarily review the core themes with brief examples of the themes and sub-themes included in the core areas. The core themes are presented below.

I. Constructive communication and rich interaction with significant others

This theme contains the narratives of how helpful social interaction and dialogue were with coaches and teammates within sport as well as partners, family, and friends outside sport. The helpfulness of teammates is illustrated in the following: “At the same time, several other injured players have been here [rehab center]. We have exercised together with our physiotherapist…We have trained a lot together. I think it has helped us very much.” Still another player said “I have received a lot of support from the girls on the team. They have encouraged me a lot, it feels good … Supportive teammates are always important; they help me to think about other things.”

Some examples of the support from coaches were: “Our coach has been really good; he has listened to me and often asked how I felt and so on” and “The coach pushed me to train harder on my own. It was great to feel that engagement from him.”

Outside sport, players mentioned that everyday life was filled with positive interactions with others who encouraged and supported the rehabilitation processes. The experiences of communication with very close persons were especially noteworthy. As one player put it, “My Mom has been like a rock in a storm. She has always been there and been attentive to me, not necessarily giving loads of advice and that kind of thing; she has just been available.”

II. Strong belief in the importance and efficacy of one’s own actions

Different kinds of personality-related factors for helping injured players cope effectively during rehabilitation emerged in the analysis. Numerous players had positive, determined attitudes, yet at the same time maintained a mindful stance under the core theme *Strong belief in the importance and efficacy of one’s own actions*. Tolerance, persistence, acceptance, and life satisfaction were all features of this core theme.

Some players even expressed attitudes of endurance in preparation for the rehabilitation to come, sometimes based on a previous positive experience of recuperation from long-term injury. One player said, “I didn’t take it very hard. When I received the diagnosis, I was prepared. I had a positive attitude to the healing process and future opportunities to handle the injury.” Yet another player said, “It has been a process to understand, to permit myself to be sad. I have accepted this now.” For some players, there was acceptance and even satisfaction with their current life situations, coupled with an awareness of their inner potential. The strength of this acceptance (and self-efficacy) is evident in the following quote, “It’s really sad that it happened, but now I have new, bright perspectives and have received answers to many of my questions. This process has been important to me and now I am positive that I will play again.” Some players exhibited a high acceptance of their situations of being ACL-injured, which helped them endure the long rehabilitation period with confidence. One player reflected on the potential the injury generated, helping her understand her present life situation:

“I don’t know. I was probably prepared for the operation and a long rehabilitation. I was organized already from the start. I told my physiotherapist to book a time with the doctor to arrange for the operation. Everything was progressing as it was supposed to. No problems at all.”

III. Ability to set reasonable goals

Under this core theme, a number of players appeared to be very focused and demonstrated strong goal-oriented behaviors during rehabilitation, with high self-generated motivation and clear plans for the future. Most of the players had already experienced successful rehabilitation from previous injuries. The following quote encapsulates how positive past rehabilitation experiences helped with their current situations:

“You should really take the time you need, use all your contacts and not be afraid to step on some toes. I have learned this from previous injury situations. We are professionals and because of that you should impose high demands on the resources in your environment and surroundings.”

In much the same way, another player stated, “The ACL injury and the rehabilitation afterwards felt like a piece of cake. I remember I thought like that the last time I was severely injured”.

One issue that repeatedly arose during the interviews was that players made plans and set clear goals for the future during rehabilitation. For instance, one player stated that, “My outlook and goal is to be back in 6 months after the operation … we play Champions League then. I will be physically and mentally ready at that time”.

## Discussion

The main result of the present study, describing resilient athletes’ experiences of rehabilitation after ACL injuries, is that we identified three core themes representing psychosocial factors that appeared to help players cope with rehabilitation: constructive communication and rich interaction with significant others, a strong belief in the importance and efficacy of one’s own actions, and an ability to set reasonable goals.

Analyzing the results of the narratives of the resilient players, it is striking how most of the players emphasized the adaptive and combined effects of supportive communications and interaction with significant persons both inside and outside their sport. Furthermore, it is also evident from the literature that a substantial part of previous research has focused on intra-individual resilience factors such as self-efficacy [8] and, to a lesser extent, on inter-individual factors such as beneficial communication with the head coach and other supportive persons during rehabilitation [11]. Although much of the attention of coaches, sports medicine team members, and sport psychologists is focused on preparing athletes for competition, this attention often vanishes when an athlete is injured. Some players in the present study also reported feelings of being excluded, neglected, or of little importance due to their physical incapacity. Despite these often observed phenomena, the resilient players appeared to benefit from interacting and constructively communicating with important persons such as parents, head coaches, teammates, and friends inside and outside the context of their sport. Some players also talked about the positive challenges the rehabilitation period brought in terms of having the opportunity to connect socially with networks of teammates and friends. These positive social features of rehabilitation processes have adaptive qualities and parallel previous research findings [10, 11].

The resilient players were also characterized by having high beliefs in their own actions, including being success-oriented and having positive attitudes as they faced their rehabilitation periods. In many ways, they matched past profiles in previous studies on athletes who have experienced successful rehabilitation after severe sports injuries [15, 16]. Personality features, such as resilience and stamina, are important psychosocial factors in the long-term management of a severe injury, usually driven by strong self-efficacy beliefs. In line with this finding, these resilient players appear not only to accept their injuries in positive ways but also to exhibit a sense of balance and satisfaction, managing challenging situations effectively. The players appeared to regard their situations as being controllable and exhibited rehabilitation behaviors that were clearly adaptive and oriented on the future. These strong self-efficacy beliefs warrant further investigation in the light of the knowledge provided to sports medicine teams when it comes to learning more about important psychosocial factors during the rehabilitation of elite female athletes.

The third core theme relates to the ability to organize oneself and plan for future activities using goals during the rehabilitation period. In part, this ability relates to the particularly relevant concept of dispositional optimism (as compared to optimistic behavior). Scheier et al., [26] the originators of this concept, defined it as representing a general expectation that good, rather than bad, outcomes will occur. These general expectations are believed to positively influence health because they tend to determine the extent to which an individual is willing to initiate health-oriented behaviors and persist with those behaviors when facing setbacks and other difficulties. Scheier et al.’s dispositional optimists tended to have more positive expectations before, during, and after surgery than those who did not obtain high scores for dispositional optimism. More specifically, dispositional optimists were found to make plans and set goals for recovery to a greater extent than pessimists [27], in much the same ways as the players in the current study. Another way to discuss the core theme of ability to set reasonable goals is related to the biopsychosocial model of Wiese-Bjornstal [28]. Research involving this model found that athletes who successfully returned to sport were characterized as having low re-injury anxiety and were more experienced and established athletes [29]. This line of research also revealed a positive relationship between goal-setting and adherence, which in turn yielded a positive relationship with the outcomes of the rehabilitation for ACL-injured athletes in the present study.

### Methodological considerations

One methodological issue to consider is that some players’ memory recall may have been inaccurate due to the fact that, on average, 6 months passed between the interviews and the injury dates, but the relatively high level of agreement between the findings and previous research speaks to the credibility of the results. One important sampling issue is that the resilient players played for/represented several different teams. It is therefore not possible to state definitively that the resilient players were predominantly located in supportive, constructive, and positively oriented teams, or had coaching staff sensitized to rehabilitation difficulties, or came from successful clubs. Another methodological consideration is whether adequate and quality data were collected to support the study (data saturation). It appears that the players’ narratives indicated behaviors that closely matched the dimensions of resilience, which include for instance self-efficacy, an ability to engage support and help, and learning from difficulties, characteristics often mentioned in the literature [14, 15]. Another related and important consideration is about the amount of data necessary to answer our research question in a credible way [30]. It is our belief that the data were sufficient to deal with the focus of our research, i.e., resilient players who exhibit self-reliance and engage in resilience-related behaviors. We feel confident in how well data and processes of analysis address the intended focus, despite the complexity of the phenomena under study.

Also, another methodological issue important to reflect on is related to the selection of narrative constructivism as the theoretical foundation for the study. The reason for selecting this approach was that it emphasizes the importance of considering a person’s story as a reflection of his or her identity, emotions, and understanding of the past, present, and future [31]. Because the aim of the study was to capture the players’ experiences in relation to the situation of being injured, we find this approach especially suitable.

Finally, the focus of the study is not the rehabilitation speed or readiness for play but rather individual experiences of rehabilitation following the reconstruction of a first-time ACL injury and the factors that contribute to a resilient, successful rehabilitation. Because the approach is idiographic, no causal relationships could be claimed between the variables and the group of interest. Future studies, tentatively based on a biospsychosocial perspective, are therefore recommended to collect combinations of different type of data to provide further evidence to support the need to capture psychosocial data on injured athletes. Such evidence can guide the development of effective techniques that will facilitate rehabilitation.

### Clinical implications

Building on the results of the present study, some practical suggestions based on adaptive behaviors of ACL-injured elite female football players during rehabilitation from ACL reconstructive surgery can be made. In the communication between the medical team and the injured player, it seems essential that both partners jointly draw up a plan for physical and mental recovery, in preparation for successful rehabilitation. Social support, in terms of communication and rich interaction, was a central theme in the present study, but curiously enough interaction with medical team was rarely mentioned. We can, however, on the strength of this theme put forward suggestions for medical team. This means that the medical team have an important mission to maintain constructive communication and rich interaction throughout the entire rehabilitation period. This implies not leaving the injured players isolated and outside the team (an *inclusive* tactic that is also the task of teammates and coaches). Also, the medical team should aim to help the players increase their self-efficacy by acknowledging and reinforcing progress in the rehabilitation process, for example. A player’s self-efficacy is not solely held within the player and it can be increased or decreased depending on the social context and the contingencies of reinforcement and punishment in the sport and rehabilitation environments. It seems equally important for the medical team to encourage injured players to set daily goals for healing and improvement, as well as long-term goals for recovery. For the injured players, having experienced prior successful rehabilitations and thus having confidence and trust in their recovery, it is recommended that they take advantage of the time out as an opportunity to rest and reflect. Finally, for the injured players, it is suggested that they continuously take advantage of the social support systems that exist both inside and outside sport (e.g., family, teammates, and medical team). This support appears to positively influence the sometimes long and arduous period that usually occurs during injury rehabilitation, or, as one player said of her physiotherapist and fellow injured athletes, “We have trained a lot together. I think it has helped us very much”.

## Conclusion

It is concluded that the findings of the present study holds unique qualities resulting in resilient behavior during rehabilitation. Recommendations for medical teams are to focus foremost on supporting communication, self-efficacy, and goal-setting during the rehabilitation process of post ACL-reconstruction.

## Abbreviation

ACL, anterior cruciate ligament
